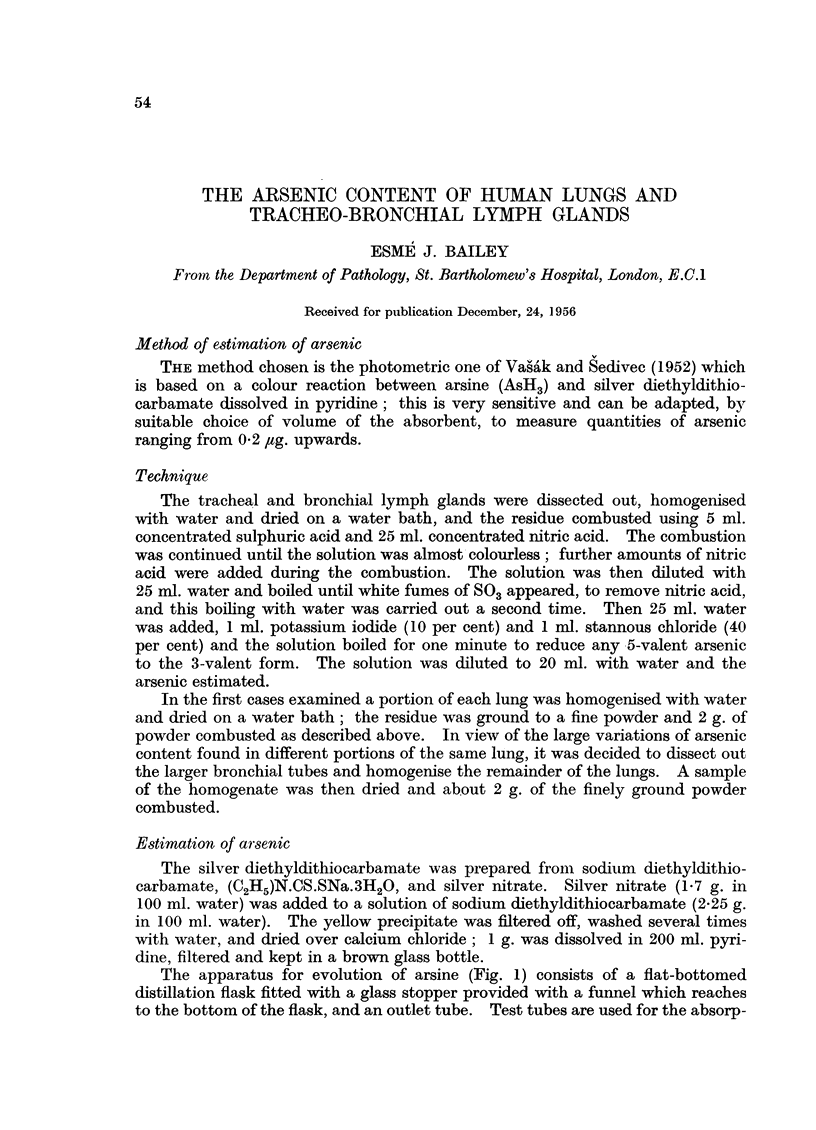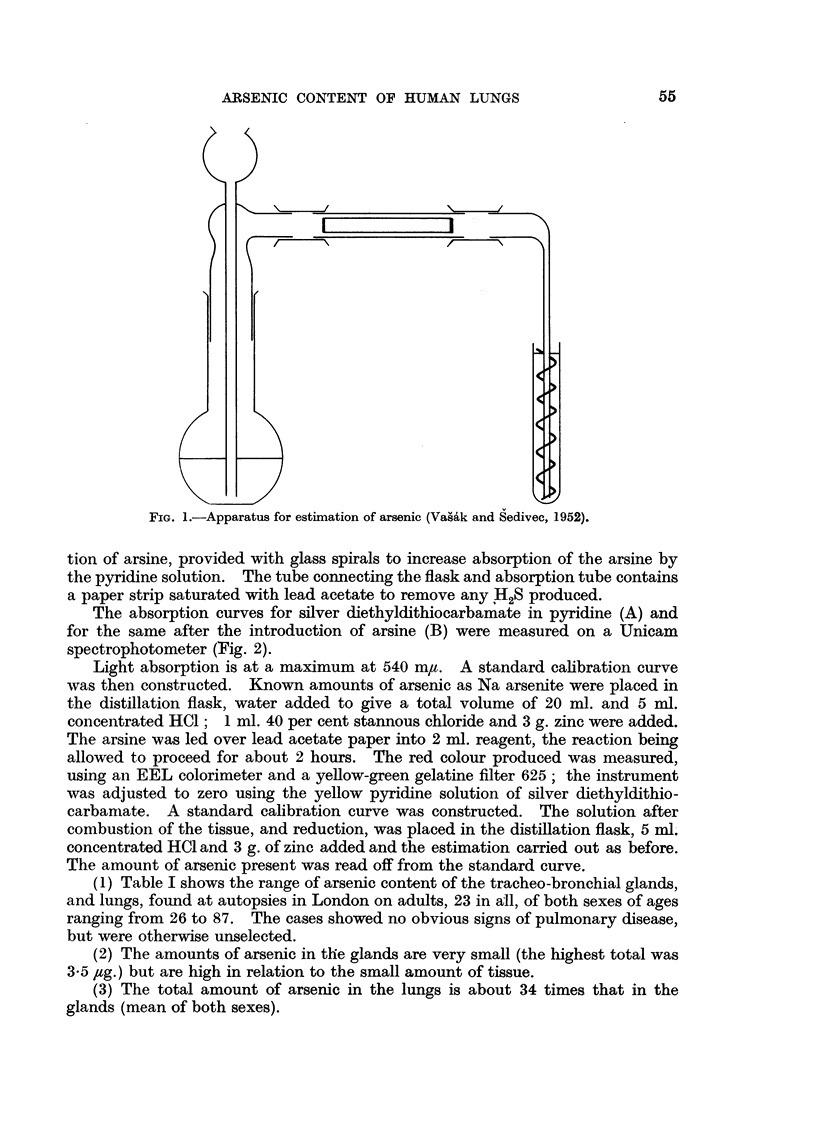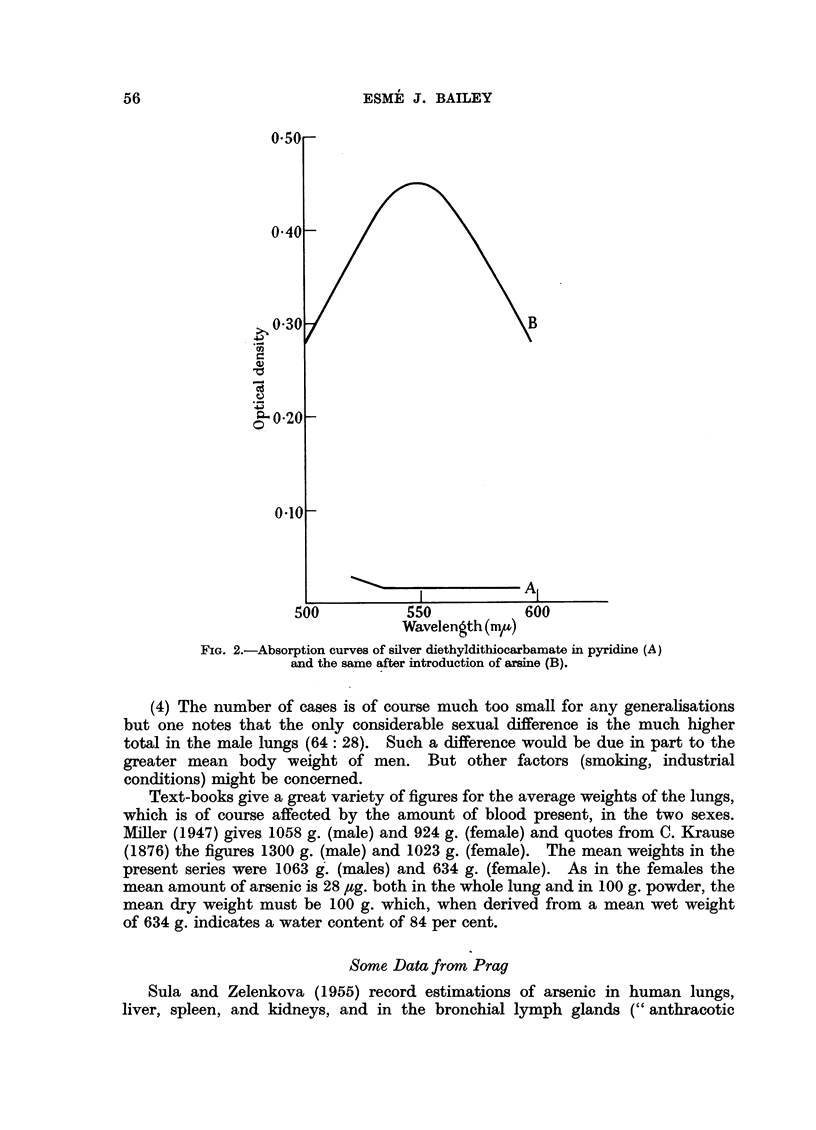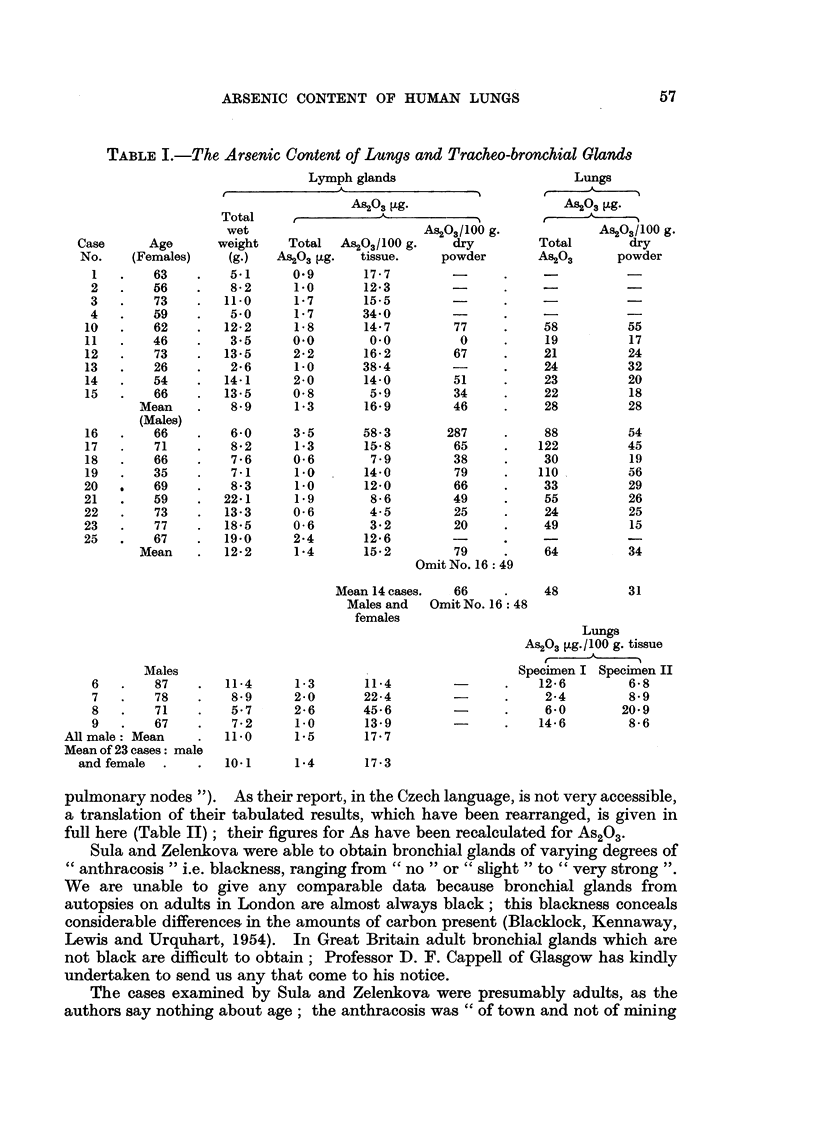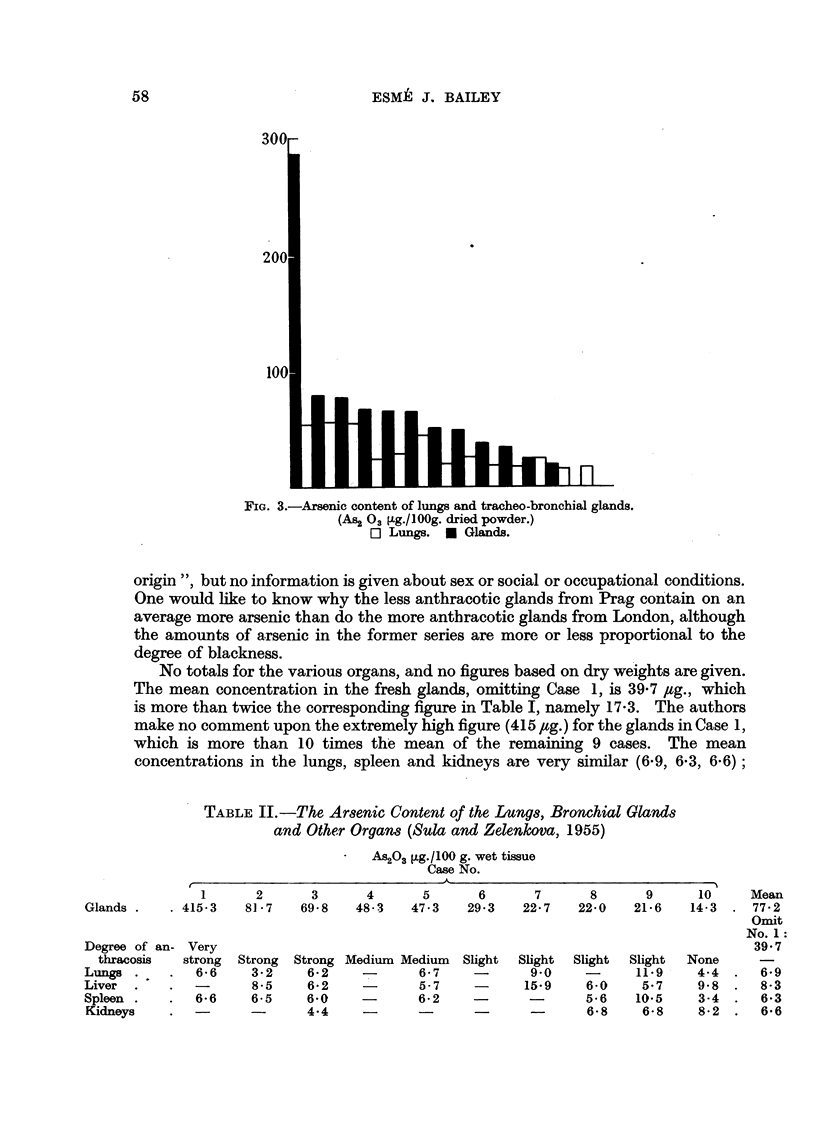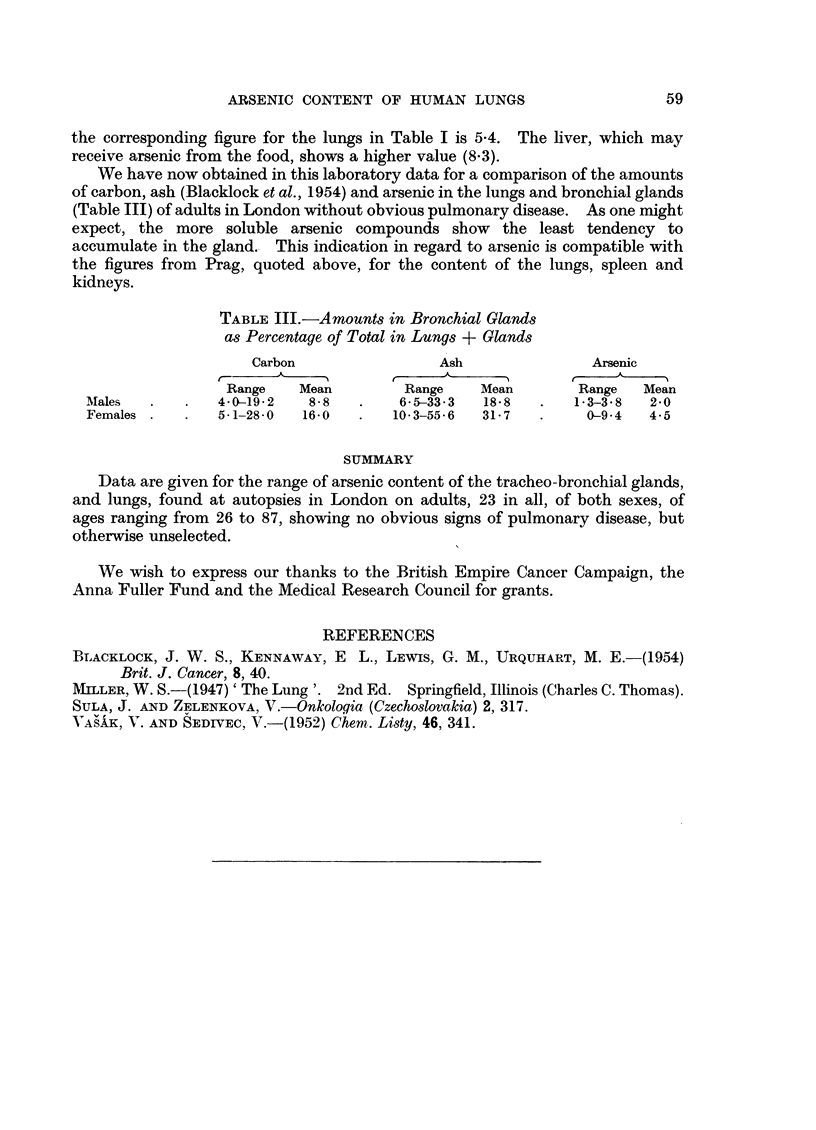# The Arsenic Content of Human Lungs and Tracheo-Bronchial Lymph Glands

**DOI:** 10.1038/bjc.1957.8

**Published:** 1957-03

**Authors:** Esmé J. Bailey


### THE ARSENIC CONTENT OF HUMAN LUNGS AND TRACHEO-BRONCHIAL LYMPH GLANDS

ESMÉ J. BAILEY

*From the Department of Pathology, St. Bartholomew's Hospital, London, E.C.*1

Received for publication December, 24, 1956

### *Method of estimation of arsenic*

THE method chosen is the photometric one of Vašák and Šedivec (1952) which is based on a colour reaction between arsine ($AsH_3$) and silver diethyldithiocarbamate dissolved in pyridine ; this is very sensitive and can be adapted, by suitable choice of volume of the absorbent, to measure quantities of arsenic ranging from 0·2 $\mu$g. upwards.

### *Technique*

The tracheal and bronchial lymph glands were dissected out, homogenised with water and dried on a water bath, and the residue combusted using 5 ml. concentrated sulphuric acid and 25 ml. concentrated nitric acid. The combustion was continued until the solution was almost colourless ; further amounts of nitric acid were added during the combustion. The solution was then diluted with 25 ml. water and boiled until white fumes of $SO_3$ appeared, to remove nitric acid, and this boiling with water was carried out a second time. Then 25 ml. water was added, 1 ml. potassium iodide (10 per cent) and 1 ml. stannous chloride (40 per cent) and the solution boiled for one minute to reduce any 5-valent arsenic to the 3-valent form. The solution was diluted to 20 ml. with water and the arsenic estimated.

In the first cases examined a portion of each lung was homogenised with water and dried on a water bath ; the residue was ground to a fine powder and 2 g. of powder combusted as described above. In view of the large variations of arsenic content found in different portions of the same lung, it was decided to dissect out the larger bronchial tubes and homogenise the remainder of the lungs. A sample of the homogenate was then dried and about 2 g. of the finely ground powder combusted.

### *Estimation of arsenic*

The silver diethyldithiocarbamate was prepared from sodium diethyldithiocarbamate, $(C_2H_5)N.CS.SNa.3H_2O$, and silver nitrate. Silver nitrate (1·7 g. in 100 ml. water) was added to a solution of sodium diethyldithiocarbamate (2·25 g. in 100 ml. water). The yellow precipitate was filtered off, washed several times with water, and dried over calcium chloride ; 1 g. was dissolved in 200 ml. pyridine, filtered and kept in a brown glass bottle.

The apparatus for evolution of arsine (Fig. 1) consists of a flat-bottomed distillation flask fitted with a glass stopper provided with a funnel which reaches to the bottom of the flask, and an outlet tube. Test tubes are used for the absorp-

FIG. 1.—Apparatus for estimation of arsenic (Vašák and Šedivec, 1952).

tion of arsine, provided with glass spirals to increase absorption of the arsine by the pyridine solution. The tube connecting the flask and absorption tube contains a paper strip saturated with lead acetate to remove any $H_2S$ produced.

The absorption curves for silver diethyldithiocarbamate in pyridine (A) and for the same after the introduction of arsine (B) were measured on a Unicam spectrophotometer (Fig. 2).

Light absorption is at a maximum at 540 m$\mu$. A standard calibration curve was then constructed. Known amounts of arsenic as Na arsenite were placed in the distillation flask, water added to give a total volume of 20 ml. and 5 ml. concentrated HCl ; 1 ml. 40 per cent stannous chloride and 3 g. zinc were added. The arsine was led over lead acetate paper into 2 ml. reagent, the reaction being allowed to proceed for about 2 hours. The red colour produced was measured, using an EEL colorimeter and a yellow-green gelatine filter 625 ; the instrument was adjusted to zero using the yellow pyridine solution of silver diethyldithiocarbamate. A standard calibration curve was constructed. The solution after combustion of the tissue, and reduction, was placed in the distillation flask, 5 ml. concentrated HCl and 3 g. of zinc added and the estimation carried out as before. The amount of arsenic present was read off from the standard curve.

(1) Table I shows the range of arsenic content of the tracheo-bronchial glands, and lungs, found at autopsies in London on adults, 23 in all, of both sexes of ages ranging from 26 to 87. The cases showed no obvious signs of pulmonary disease, but were otherwise unselected.

(2) The amounts of arsenic in the glands are very small (the highest total was 3·5 $\mu$g.) but are high in relation to the small amount of tissue.

(3) The total amount of arsenic in the lungs is about 34 times that in the glands (mean of both sexes).

Fig. 2.—Absorption curves of silver diethyldithiocarbamate in pyridine (A) and the same after introduction of arsine (B).

(4) The number of cases is of course much too small for any generalisations but one notes that the only considerable sexual difference is the much higher total in the male lungs (64 : 28). Such a difference would be due in part to the greater mean body weight of men. But other factors (smoking, industrial conditions) might be concerned.

Text-books give a great variety of figures for the average weights of the lungs, which is of course affected by the amount of blood present, in the two sexes. Miller (1947) gives 1058 g. (male) and 924 g. (female) and quotes from C. Krause (1876) the figures 1300 g. (male) and 1023 g. (female). The mean weights in the present series were 1063 g. (males) and 634 g. (female). As in the females the mean amount of arsenic is 28 μg. both in the whole lung and in 100 g. powder, the mean dry weight must be 100 g. which, when derived from a mean wet weight of 634 g. indicates a water content of 84 per cent.

*Some Data from Prag*

Sula and Zelenkova (1955) record estimations of arsenic in human lungs, liver, spleen, and kidneys, and in the bronchial lymph glands (" anthracotic

TABLE I.—*The Arsenic Content of Lungs and Tracheo-bronchial Glands*

| Case No. | Age (Females) | Lymph glands: Total wet weight (g.) | Lymph glands $As_2O_3$ µg.: Total $As_2O_3$ µg. | Lymph glands $As_2O_3$ µg.: $As_2O_3$/100 g. tissue. | Lymph glands $As_2O_3$ µg.: $As_2O_3$/100 g. dry powder | Lungs $As_2O_3$ µg.: Total $As_2O_3$ | Lungs $As_2O_3$ µg.: $As_2O_3$/100 g. dry powder |
|---|---|---|---|---|---|---|---|
| 1 | 63 | 5·1 | 0·9 | 17·7 | — | — | — |
| 2 | 56 | 8·2 | 1·0 | 12·3 | — | — | — |
| 3 | 73 | 11·0 | 1·7 | 15·5 | — | — | — |
| 4 | 59 | 5·0 | 1·7 | 34·0 | — | — | — |
| 10 | 62 | 12·2 | 1·8 | 14·7 | 77 | 58 | 55 |
| 11 | 46 | 3·5 | 0·0 | 0·0 | 0 | 19 | 17 |
| 12 | 73 | 13·5 | 2·2 | 16·2 | 67 | 21 | 24 |
| 13 | 26 | 2·6 | 1·0 | 38·4 | — | 24 | 32 |
| 14 | 54 | 14·1 | 2·0 | 14·0 | 51 | 23 | 20 |
| 15 | 66 | 13·5 | 0·8 | 5·9 | 34 | 22 | 18 |
| | Mean | 8·9 | 1·3 | 16·9 | 46 | 28 | 28 |
| | (Males) | | | | | | |
| 16 | 66 | 6·0 | 3·5 | 58·3 | 287 | 88 | 54 |
| 17 | 71 | 8·2 | 1·3 | 15·8 | 65 | 122 | 45 |
| 18 | 66 | 7·6 | 0·6 | 7·9 | 38 | 30 | 19 |
| 19 | 35 | 7·1 | 1·0 | 14·0 | 79 | 110 | 56 |
| 20 | 69 | 8·3 | 1·0 | 12·0 | 66 | 33 | 29 |
| 21 | 59 | 22·1 | 1·9 | 8·6 | 49 | 55 | 26 |
| 22 | 73 | 13·3 | 0·6 | 4·5 | 25 | 24 | 25 |
| 23 | 77 | 18·5 | 0·6 | 3·2 | 20 | 49 | 15 |
| 25 | 67 | 19·0 | 2·4 | 12·6 | — | — | — |
| | Mean | 12·2 | 1·4 | 15·2 | 79<br>Omit No. 16 : 49 | 64 | 34 |
| | | | | Mean 14 cases. Males and females | 66<br>Omit No. 16 : 48 | 48 | 31 |
| | | | | | | Lungs $As_2O_3$ µg./100 g. tissue: Specimen I | Lungs $As_2O_3$ µg./100 g. tissue: Specimen II |
| | Males | | | | | | |
| 6 | 87 | 11·4 | 1·3 | 11·4 | — | 12·6 | 6·8 |
| 7 | 78 | 8·9 | 2·0 | 22·4 | — | 2·4 | 8·9 |
| 8 | 71 | 5·7 | 2·6 | 45·6 | — | 6·0 | 20·9 |
| 9 | 67 | 7·2 | 1·0 | 13·9 | — | 14·6 | 8·6 |
| All male : Mean | | 11·0 | 1·5 | 17·7 | | | |
| Mean of 23 cases : male and female | | 10·1 | 1·4 | 17·3 | | | |

pulmonary nodes "). As their report, in the Czech language, is not very accessible, a translation of their tabulated results, which have been rearranged, is given in full here (Table II) ; their figures for As have been recalculated for $As_2O_3$.

Sula and Zelenkova were able to obtain bronchial glands of varying degrees of " anthracosis " i.e. blackness, ranging from " no " or " slight " to " very strong ". We are unable to give any comparable data because bronchial glands from autopsies on adults in London are almost always black ; this blackness conceals considerable differences in the amounts of carbon present (Blacklock, Kennaway, Lewis and Urquhart, 1954). In Great Britain adult bronchial glands which are not black are difficult to obtain ; Professor D. F. Cappell of Glasgow has kindly undertaken to send us any that come to his notice.

The cases examined by Sula and Zelenkova were presumably adults, as the authors say nothing about age ; the anthracosis was " of town and not of mining

FIG. 3.—Arsenic content of lungs and tracheo-bronchial glands. ($As_2O_3$ μg./100g. dried powder.) □ Lungs. ■ Glands.

origin ", but no information is given about sex or social or occupational conditions. One would like to know why the less anthracotic glands from Prag contain on an average more arsenic than do the more anthracotic glands from London, although the amounts of arsenic in the former series are more or less proportional to the degree of blackness.

No totals for the various organs, and no figures based on dry weights are given. The mean concentration in the fresh glands, omitting Case 1, is 39·7 μg., which is more than twice the corresponding figure in Table I, namely 17·3. The authors make no comment upon the extremely high figure (415 μg.) for the glands in Case 1, which is more than 10 times the mean of the remaining 9 cases. The mean concentrations in the lungs, spleen and kidneys are very similar (6·9, 6·3, 6·6);

TABLE II.—*The Arsenic Content of the Lungs, Bronchial Glands and Other Organs* (*Sula and Zelenkova*, 1955)

$As_2O_3$ μg./100 g. wet tissue

| | Case No. | | | | | | | | | | |
|---|---|---|---|---|---|---|---|---|---|---|---|
| | 1 | 2 | 3 | 4 | 5 | 6 | 7 | 8 | 9 | 10 | Mean |
| Glands | 415·3 | 81·7 | 69·8 | 48·3 | 47·3 | 29·3 | 22·7 | 22·0 | 21·6 | 14·3 | 77·2 Omit No. 1: 39·7 |
| Degree of anthracosis | Very strong | Strong | Strong | Medium | Medium | Slight | Slight | Slight | Slight | None | — |
| Lungs | 6·6 | 3·2 | 6·2 | — | 6·7 | — | 9·0 | — | 11·9 | 4·4 | 6·9 |
| Liver | — | 8·5 | 6·2 | — | 5·7 | — | 15·9 | 6·0 | 5·7 | 9·8 | 8·3 |
| Spleen | 6·6 | 6·5 | 6·0 | — | 6·2 | — | — | 5·6 | 10·5 | 3·4 | 6·3 |
| Kidneys | — | — | 4·4 | — | — | — | — | 6·8 | 6·8 | 8·2 | 6·6 |

the corresponding figure for the lungs in Table I is 5·4. The liver, which may receive arsenic from the food, shows a higher value (8·3).

We have now obtained in this laboratory data for a comparison of the amounts of carbon, ash (Blacklock *et al.*, 1954) and arsenic in the lungs and bronchial glands (Table III) of adults in London without obvious pulmonary disease. As one might expect, the more soluble arsenic compounds show the least tendency to accumulate in the gland. This indication in regard to arsenic is compatible with the figures from Prag, quoted above, for the content of the lungs, spleen and kidneys.

TABLE III.—*Amounts in Bronchial Glands as Percentage of Total in Lungs + Glands*

| | Carbon | | Ash | | Arsenic | |
|---|---|---|---|---|---|---|
| | Range | Mean | Range | Mean | Range | Mean |
| Males | 4·0–19·2 | 8·8 | 6·5–33·3 | 18·8 | 1·3–3·8 | 2·0 |
| Females | 5·1–28·0 | 16·0 | 10·3–55·6 | 31·7 | 0–9·4 | 4·5 |

SUMMARY

Data are given for the range of arsenic content of the tracheo-bronchial glands, and lungs, found at autopsies in London on adults, 23 in all, of both sexes, of ages ranging from 26 to 87, showing no obvious signs of pulmonary disease, but otherwise unselected.

We wish to express our thanks to the British Empire Cancer Campaign, the Anna Fuller Fund and the Medical Research Council for grants.

### REFERENCES

BLACKLOCK, J. W. S., KENNAWAY, E L., LEWIS, G. M., URQUHART, M. E.—(1954) *Brit. J. Cancer*, **8**, 40.
MILLER, W. S.—(1947) ' The Lung '. 2nd Ed. Springfield, Illinois (Charles C. Thomas).
SULA, J. AND ZELENKOVA, V.—*Onkologia* (*Czechoslovakia*) **2**, 317.
VAŠÁK, V. AND ŠEDIVEC, V.—(1952) *Chem. Listy*, **46**, 341.